# Supplementary material for: Metabolomic Profiling of Citrus unshiu during Different Stages of Fruit Development
Source: Plants (Basel). 2022 Apr 1;11(7):967. doi: 10.3390/plants11070967 (PMC9002680; doi:10.3390/plants11070967)
Supplement: Supplementary file 1 [file plants-11-00967-s001.zip › plants-1623296-supplementary.pdf]

Supplementary materials

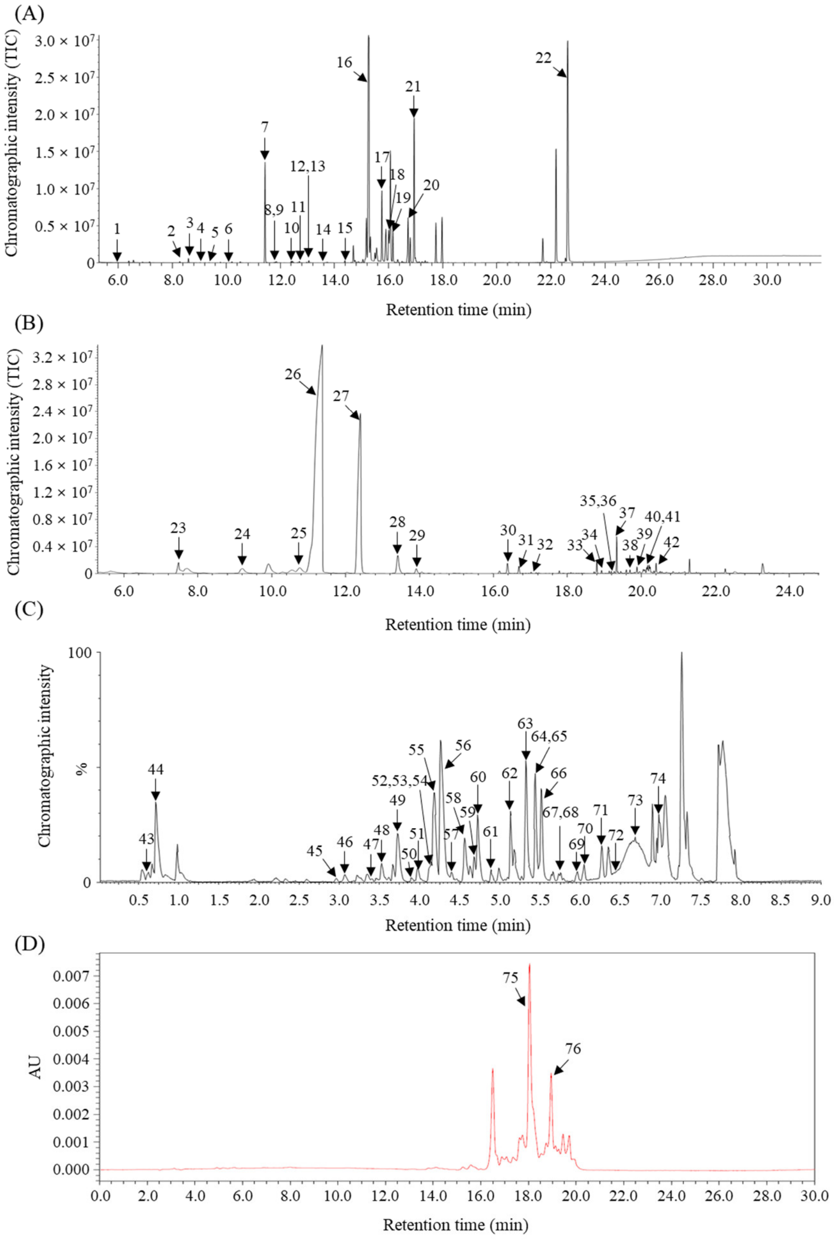

Figure S1. Representative chromatograms of citrus flesh metabolites analyzed by GC-MS (A: non-volatile compounds and B: volatile compounds), UPLC-QTOF MS (C), and HPLC (D). 1, alanine; 2, serine; 3, phosphoric acid; 4, proline; 5, propionic acid; 6, threonine; 7, malic acid; 8, glutamic acid; 9, aspartic acid; 10, threonic acid; 11, glutaric acid; 12, glutamine; 13, suberyl glycine; 14, asparagine; 15, aconitic acid; 16, citric acid; 17, quinic acid; 18, fructose; 19, mannose; 20, inositol; 21, glucose; 22, sucrose; 23, α-pinene; 24, β-pinene; 25, α-terpinene; 26, limonene; 27, γ-terpinene; 28, α-terpinolene; 29, linalool; 30, 4-terpineol; 31, α-terpineol; 32, decanal; 33, δ-elemene; 34, α-cubebene; 35, texanol; 36, α-copaene; 37, β-elemene; 38, γ-elemene; 39, α-humulene; 40, germacrene D; 41, α-farnesene; 42, δ-cadinene; 43, arginine; 44, stachydrine; 45, phenylalanine; 46, tryptophan; 47, quercetin triglucoside; 48, saponarin; 49, quercetin triglucoside derivative; 50, kaempferol triglucoside; 51, hesperidin triglucoside; 52, kaempferil-3-rutinoside; 53, zapoterin; 54, isorhamnetin-3-O-rutinoside; 55, narirutin; 56, hesperidin; 57, xalogranatin K; 58, didymin; 59, nomilin; 60, cyclonatsudamine A; 61, Gly-Ile-Pro-Tyr-Ile-Ala-Ala; 62, limonin; 63, nobiletin; 64, phytosphingosine; 65, methoxynobiletin; 66, tangeretin; 67, LPE(C18:3); 68, LPC(C18:3); 69, LPC(C16:1); 70, LPC(C18:2); 71, LPC(C16:0); 72, LPE(C20:0); 73, LPC(C18:0); 74, pheophorbide A; 75, β-cryptoxanthin; 76, β-carotene.

Table S1. Morphological characteristics of *C. unshiu* during fruit development

	Weight (g)			Size (cm)		Color			
	Flesh	Peel	Whole fruit	Width	Length	*L**	*a**	*b**	CCI
Aug. 1	21.86 ± 2.63^{e}	9.51 ± 1.32^{d}	31.37 ± 3.79^{ae}	3.47 ± 1.92^{e}	4.16 ± 1.68^{d}	40.77 ± 2.83^{c}	-13.11 ± 1.77^{c}	19.66 ± 3.98^{b}	-16.66 ± 1.93^{c}
Aug. 31	41.51 ± 5.88^{d}	11.69 ± 1.99^{c}	53.20 ± 7.36^{d}	4.96 ± 3.13^{d}	3.94 ± 2.04^{d}	42.75 ± 2.99^{b}	-13.51 ± 2.14^{c}	21.27 ± 3.86^{b}	-15.03 ± 1.59^{b}
Sep. 14	63.17 ± 8.62^{c}	19.03 ± 3.37^{b}	82.20 ± 9.56^{c}	5.75 ± 2.01^{c}	4.69 ± 3.15^{c}	41.13 ± 3.29^{c}	-13.13 ± 1.99^{c}	20.11 ± 4.00^{b}	-16.20 ± 2.18^{c}
Oct. 15	77.08 ± 6.09^{b}	18.23 ± 1.91^{b}	95.32 ± 6.96^{ab}	6.14 ± 2.01^{b}	4.82 ± 1.64^{b}	62.00 ± 3.21^{a}	2.24 ± 5.21^{b}	36.19 ± 2.33^{a}	0.88 ± 2.32^{b}
Nov. 16	88.27 ± 9.76^{a}	23.19 ± 3.16^{a}	111.45 ± 11.51^{a}	6.44 ± 2.71^{a}	5.20 ± 2.24^{a}	60.86 ± 1.87^{a}	25.68 ± 2.19^{a}	36.65 ± 1.23^{a}	11.59 ± 1.54^{a}

*L**, lightness; *a**, redness; *b**, yellowness; CCI, citrus color index.

Different letters in each column indicate significant differences by Duncan's test ($p < 0.05$).

Table S2. Identification of major metabolites by GC-MS

	RT	Compounds
Non-volatile compounds	5.99	alanine
	8.29	serine
	8.60	phosphoric acid
	8.84	proline
	9.38	propionic acid
	10.15	threonine
	11.43	malic acid
	11.77	glutamic acid
	11.82	aspartic acid
	12.40	threonic acid
	12.45	glutaric acid
	12.98	glutamine
	13.15	suberyl glycine
	13.56	asparagine
	14.38	aconitic acid
	15.27	citric acid
	15.75	quinic acid
	16.01	fructose
	16.16	mannose
	16.73	inositol
	16.95	glucose
	22.62	sucrose
Volatile compounds	7.75	α-pinene
	9.24	β-pinene
	10.77	α-terpinene
	11.47	limonene
	12.46	γ-terpinene
	13.44	α-terpinolene
	13.98	linalool
	16.39	4-terpineol
	16.70	α-terpineol
	17.03	decanal
	18.81	δ-elemene
	18.93	α-cubebene
	19.15	texanol
	19.20	α-copaene
	19.35	β-elemene
	19.70	γ-elemene
	19.90	α-humulene
	20.11	germacrene D
	20.25	α-farnesene
	20.41	δ-cadinene

RT, retention time; VIP, variable importance in the protection.

Table S3. Identification of major metabolites by UPLC-QTOF MS and HPLC

	RT	Compounds	m/z	MS fragment
UPLC-Q-TOF MS	0.61	arginine	175.1192	70, 158, 116
	0.72	stachydrine	144.1022	116, 184, 70, 102, 58
	2.59	phenylalanine	166.0864	102, 103
	3.06	tryptophan	205.0973	188, 146, 118
	3.40	quercetin triglucoside 1	757.2189	611, 303
	3.52	saponarin	595.1661	577, 559
	3.73	quercetin triglucoside 2	757.2179	287, 449, 303, 465
	3.90	kaempferol triglucoside	741.2241	287, 449
	3.92	hesperetin triglucoside	771.2336	611, 317, 287, 595, 479
	4.07	kaempferol-3-rutinoside	595.1663	287, 449
	4.11	zapoterin	471.2018	425, 95
	4.15	isorhamnetin-3-O-rutinoside	625.1763	377, 285
	4.17	narirutin	581.1874	273, 419, 119
	4.26	hesperidin	611.1981	303, 449, 177
	4.40	xylogranatin K	515.2281	496, 409
	4.55	didymin	595.2028	287, 433, 559
	4.66	nomilin	515.2274	
	4.68	cyclonatsudamine A	728.3987	615, 587, 502, 474
	4.88	Gly-Ile-Pro-Tyr-Ile-Ala-Ala	704.3986	686, 668, 318, 306, 86
	5.18	limonin	471.2042	161, 95
	5.32	nobiletin	403.1380	373, 355
	5.42	phytosphingosine	318.3007	300, 282
	5.44	methoxynobiletin	433.1502	403, 418, 385
	5.51	tangeretin	373.1289	343, 325
	5.77	LPE(C18:3)	476.2770	458, 335
	5.78	LPC(C18:3)	518.3244	184, 86
	5.90	LPC(C16:1)	494.3239	476, 184
	6.02	LPC(C18:2)	520.3404	184, 86
	6.26	LPC(C16:0)	496.3402	184, 478, 86, 313
	6.45	LPE(C20:0)	510.3549	184, 492, 327
	6.72	LPC(C18:0)	524.3700	184, 506, 104
	6.98	pheophorbide A	593.2768	553, 492
HPLC	18.04	β-cryptoxanthin		
	18.95	β-carotene		

RT, retention time; VIP, variable importance in the protection; LPE, lysophosphatidylethanolamine; LPC, lysophosphatidylcholine.
